# Coaxial Dipole Array With Switching Transmit Sensitivities for Ultrahigh Field MRI

**DOI:** 10.1002/mrm.70243

**Published:** 2026-02-09

**Authors:** Dario Bosch, Georgiy A. Solomakha, Felix Glang, Martin Freudensprung, Nikolai Avdievich, Klaus Scheffler

**Affiliations:** ^1^ Magnetic Resonance Center Max Planck Institute for Biological Cybernetics Tübingen Germany; ^2^ Biomedical Magnetic Resonance University of Tübingen Tübingen Germany; ^3^ MRI Core Facility of the Medical Faculty University of Tübingen Tübingen Germany; ^4^ Institute of Biomedical Imaging Graz University of Technology Graz Austria; ^5^ Institute of Neuroradiology University Hospital Erlangen Erlangen Germany

**Keywords:** arrays, dipoles, kT‐points, pTx, RF‐shimming

## Abstract

**Purpose:**

To investigate dipole antennas with electronically switchable transmit field patterns to improve flip angle homogeneity in ultra‐high field MRI.

**Methods:**

Reconfigurable dipole elements that could produce two distinct electronically switchable $B_{1}^{+}$ field profiles were conceptualized and constructed. Eight such elements were combined into an array. Alteration of the field profiles was accomplished by modulating the currents along the dipoles using a combination of PIN diodes and lumped inductors. The behavior of these reconfigurable elements was studied in numerical electromagnetic simulations and 9.4T MRI measurements, investigating rapid switching of transmit sensitivities during excitation pulses in both single‐channel and pTx mode operation.

**Results:**

For the simulated dipole elements, modulating the current densities along the dipole's axis causes a ∼30% change of the $B_{1}^{+}$ field between superior and inferior regions of the brain. When rapidly switched during excitation pulses, this degree of freedom can improve flip angle homogeneity, e.g., by a factor of ∼2.2 for a two kT points pTx pulse. For the constructed prototype array, the switching effect was observable but weaker, causing ∼10% superior‐inferior $B_{1}^{+}$ variation.

**Conclusions:**

The proposed coaxial dipole array with switchable transmit sensitivities offers a novel degree of freedom for designing excitation pulses. The approach has the potential to improve flip angle homogeneity without necessitating an expensive increase in the number of independent transmit channels.

## 1 Introduction

In ultra‐high field MRI (UHF MRI, ≥ 7 Tesla), the transmit (Tx) radio‐frequency (RF) field ($B_{1}^{+}$) is inherently inhomogeneous due to its short wavelength relative to sample size [1, 2]. Parallel transmit (pTx) systems address this by independently driving 8–16 RF elements, enabling control of magnitude and phase per channel [1, 3], thus giving control over the interference pattern of the RF field generated by the Tx elements. This is generally referred to as “RF shimming” [4]. Unlike fixed power splitters, per‐channel amplifiers allow dynamic, slice‐wise shimming and optimized composite pulses such as spokes pulses or kT‐points, [5], where an optimized RF shim is computed for each individual sub‐pulse.

Increasing Tx channels raises degrees of freedom but is rare due to cost and complexity. With 16‐element coils on 8‐channel systems, power splitters can drive multiple elements per channel (symmetrically or asymmetrically) [6, 7]. This does not increase control flexibility, but allows for improved Tx array designs that offer, for example, larger coverage. An alternative is Tx multiplexing [8], in which fast, high‐power electronic switches connect multiple Tx elements to a single Tx channel. This method, however, adds hardware complexity, requiring robust, rapid switches and a larger number of Tx elements.

Receive (Rx) loop [9] and dipole [10, 11] elements with switchable sensitivities boost parallel imaging performance [12] and reduce noise amplification due to parallel imaging reconstruction. Additional electronic components within the Rx element allow changing the spatial distribution of its sensitivity. The sensitivities were switched back and forth during the signal acquisition phase of an MRI pulse sequence, and the Rx sensitivity states were treated like different coil images in SENSE [12] parallel imaging reconstruction.

Inspired by prior work, we extend switchable‐sensitivity concepts to transmit elements. A coaxial dipole [13, 14] has a current distribution symmetric about its feed, yielding a symmetric $B_{1}^{+}$. Adding lumped inductors between shield and center conductor at both ends preserves the symmetry. However, making one end capacitive (or open) skews the current and thus $B_{1}^{+}$ toward that end. By toggling these terminations, we obtain time‐varying transmit sensitivities that serve as extra degrees of freedom for RF pulse design.

Here we investigate the potential of such dipoles to improve flip angle homogeneity by rapidly switching sensitivities in between the sub‐pulses of both CP mode and optimized kT points excitation pulses. In addition, we perform an experimental in vivo proof‐of‐concept with a self‐built prototype eight‐element coil array. Preliminary simulation results have been presented on previous conferences [15, 16].

## 2 Methods

### 2.1 Design and Numerical Simulations of the Element

First, single dipoles were simulated to demonstrate how the $B_{1}^{+}$ field pattern changes with changing load impedance at the dipole's end. This was done in CST Studio 2021 (Dassault Systèmes, France), using the finite‐element method in the frequency domain. A single coaxial dipole was constructed from coaxial cable with a center conductor diameter of 1 mm and a shield diameter of 3.4 mm, similar to previous publications [11, 14]. Both central conductor and shield were made from copper. Inner and outer coaxial cable diameters were doubled compared to the real coaxial cable to increase the feasibility of the numerical simulations. To validate this approach, the array was simulated with the wire diameter used for construction of the array (0.5 mm) and with 1 mm diameter. The high number of mesh cells for the smaller diameter (285.8 million cells vs 92.7 million cells) made the CST simulations impractically slow. While the SAR distribution stayed similar qualitatively, the peak value increased by 13.4% for the smaller diameter. Similarly, the $B_{1}^{+}$ distribution pattern barely changed, with a slight increase in absolute $B_{1}^{+}$ for the thinner wires. The inaccuracy of the simulations was accounted for by a safety factor applied to the SAR model. The $B_{1}^{+}$ changes were considered too minor to have any effect on the practical use of the array.

**FIGURE 1:**
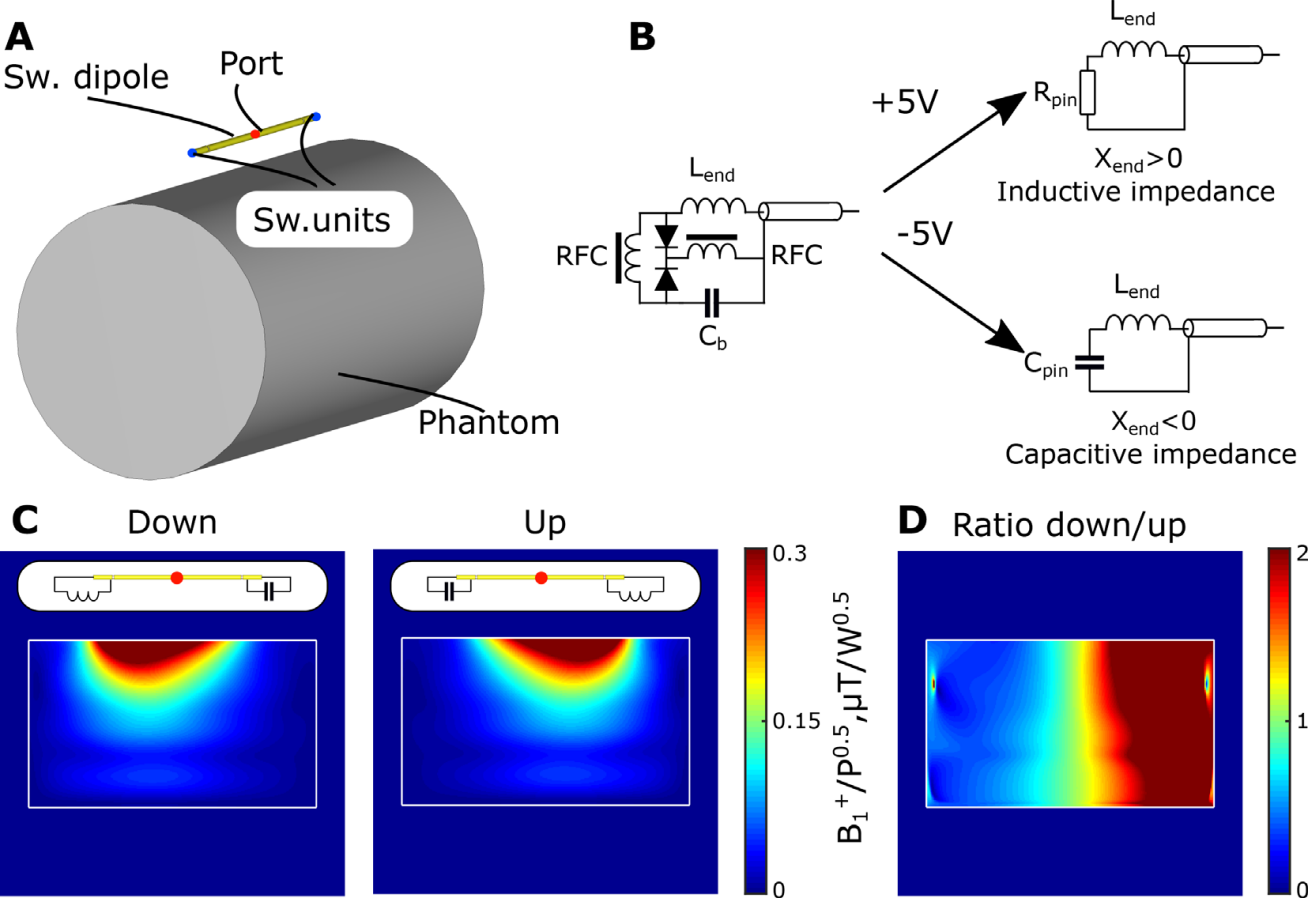
Illustration of functioning principle of the switchable dipole element. **(A)** View of the numerical model of a single switchable coaxial dipole antenna. **(B)** Switchable unit circuit for high power operation. **(C)** Numerically simulated $B_{1}^{+}$ field for “down” and “up” configuration of switchable coaxial dipole antenna. **(D)** Ratio between down and up field configuration.

The total length of the coaxial dipole was 17 cm with slots located at 20 mm distance from the dipole's ends. The dipole element was loaded to a 175 mm diameter ($D_{ph}$) and 300 mm length ($L_{ph}$) homogeneous cylindrical phantom. The dielectric properties of the phantom (ϵ = 58, σ = 0.64 S/m) correspond to averaged human brain tissues properties at the 9.4 T MRI frequency of 400 MHz. A view of the numerical model of a single switchable coaxial dipole antenna is presented in Figure 1A.

The ideal configuration with $B_{1}^{+}$ skewed to one side of an object correspond to a dipole antenna with an inductance on that end and an open circuit at the other end. Our previously used switchable unit circuit for reconfigurable receive elements [11] (PIN diode connected in series with inductance) is not suitable for operation in Tx‐mode since the negatively biased PIN diode could be opened by the high‐voltage RF pulse [17]. To prevent the forward‐ biased PIN diode from being switched off, we modified the switching circuit by adding an additional counter‐directed PIN diode. The proposed circuit of a high‐power switchable unit is presented in Figure 1B. In all numerical simulations the following parameters were used for the switching unit circuit: $L_{\text{end}}$ = 40 nH with Q‐factor of 150, $C_{\text{pin}}$ = 1.1 pF (corresponds to the capacitance of two negatively biased high‐power PIN diodes MA4P7446F‐1091 (MACOM, USA) in series), $R_{\text{pin}}$ = 1 Ω.

### 2.2 Coil Design and Simulation

**FIGURE 2:**
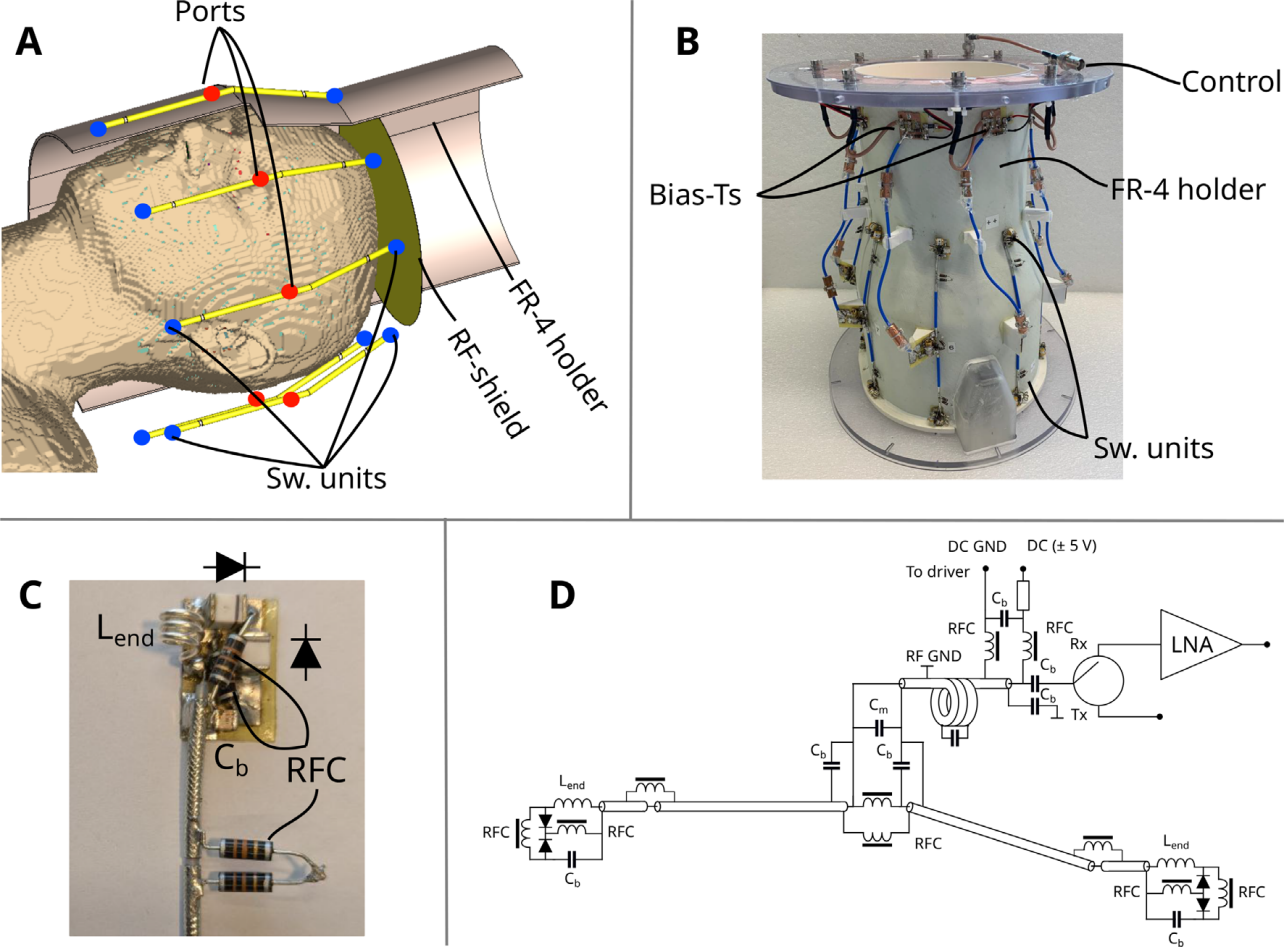
**(A)** View of the numerical model of the switchable coaxial dipole antenna array in CST Studio 2021 loaded with the Duke voxel model. **(B)** Photo of the prototype of the switchable coaxial dipole antenna array. **(C)** Enlarged photo of a switchable unit placed at the coaxial dipole end. **(D)** Circuit diagram of a switchable coaxial dipole antenna.

Following simulations of a single switchable dipole, an eight‐channel array of switchable coaxial dipole antennas was investigated numerically. For this, the finite integration technique in time‐domain was used in CST Studio 2021. The eight‐channel switchable array was mounted on a 3 mm thick FR‐4 holder. The shape of the holder was identical to the one previously used for evaluation of a non‐switchable coaxial dipole head array for 9.4 T [14]. A local RF‐shield was added at 2 cm distance from the top end of the dipoles similarly to other dipole arrays [14, 18, 19, 20] developed in our group to increase field homogeneity. A 1600 mm length copper cylinder with 640 mm diameter was added to the numerical model to mimic the RF shield (bore) of our MRI scanner. The array was loaded with multi‐tissue human models “Duke” and “Ella” (Zurich MedTech, Switzerland) with 2 mm resolution. A view of the array's numerical model loaded with the Duke voxel model is presented in Figure 2.

For comparison, the non‐switchable coaxial dipole array from work [14] was also simulated as a reference. The simulations resulted in three sets of $B_{1}^{+}$ maps: “up” and “down,” where all sensitivity profiles were shifted in the cranial/caudal direction, and the reference array's symmetrical maps. Based on the results of the numerical simulations, Q‐matrices were calculated and compressed to VOP‐matrices [21] using the modified compression algorithm from Orzada et al. [22] with an overestimation factor of 2.2. The VOP‐file for the switchable array was constructed by concatenating the Q‐matrices of the “up” and “down” states before VOP compression. This procedure treats the two states of the array equivalently to two different voxel models. This ensures that all possible SAR distributions are safely represented by the jointly compressed VOP matrices.

The switchable array was tested on the bench with a vector network analyzer. It was tuned and matched in the “up”‐configuration on a head‐and‐shoulder phantom with tissue‐like dielectric properties. The scattering‐matrix (S‐matrix) was evaluated for both the “up” and the “down” configuration. Both S‐matrices were also evaluated in simulation.

### 2.3 Numerical Pulse Optimization

As a first proof of concept, the simulated $B_{1}^{+}$ maps were used for RF pulse optimization. A homogeneous flip angle distribution of 10° throughout the brain was chosen as the optimization target. The normalized root mean square error (nRMSE) of the obtained flip angle distribution was used as a quality metric. The optimization was performed based on the spatial domain method [23] using the variable exchange method [24] to solve the magnitude‐least‐squares problem with 20 iterations and no regularization. Both single‐channel operation in circularly polarized (CP) mode and eight‐channel pTx operation were tested. For the switchable array, fast switching during the pulse was implemented by changing the coil profiles from “up” to “down” configuration after half the excitation time. In this case, magnitude and phase were optimized for each subpulse individually. The A‐Matrix of the spatial domain method thus included two columns with the respective sensitivity profile for each k‐space position, which were present at slightly different points in time.

For CP mode operation, one (reference array) or two (switchable array) global magnitudes and phases were calculated, which drove all Tx elements. For pTx operation, one or two RF shims were calculated instead. A 2‐kT point excitation [5] was also evaluated for both CP and pTx mode. Note that, compared to the conventional kT points scenario in pTx mode, which requires suitable pTx hardware to apply variable RF shims for different subpulses, gradient blips between subpulses can also be used with CP mode excitation only. In this case, only the global magnitude and phase of each subpulse are variable, which can be executed using standard single‐channel Tx hardware.

Since the focus of this work was to determine the effect of the switchable element, we tried to prevent any bias by the algorithm that determines the k‐space locations of the kT‐points. This was done by randomly generating 10 000 k‐space positions in the range $-14\;m^{-1}$ $\leq k_{x,y,z}\leq 14\;m^{-1}$ and selecting the one that gave the best performance for the given scenario as the first kT point. The second kT point was always positioned in the center of the k‐space.

### 2.4 Switchable Coaxial Dipole Array Prototype

Based on the simulation results, an eight‐channel 17 cm switchable array was constructed (Figure 2A). An optical trigger controlled by the pulse sequence was used for switching. The optical trigger was converted to a TTL‐signal and then used to control a CMOS driver to switch the PIN diodes' states by applying ± 5 V to the control input of the switchable array. The switching signal was injected to the inputs of the switchable dipole elements using home‐built bias‐tees. The dipoles were constructed using semi‐flexible non‐magnetic RG‐405 cable (Carlisle Interconnect). The dipole inputs and bias‐tees outputs were connected using the same RG‐405 cable with two floating ground cable traps [25] to prevent wave (common mode) propagation along the cable. The inputs of the bias‐tees were connected to eight panel BNC connectors (Huber+Suhner, Switzerland) mounted on a polycarbonate flange plate. A photo of a single switchable unit consisting of two high‐power PIN diodes (MA4P7446F‐1091, MACOM, USA), lumped self‐made 40 nH inductor (1.2 mm wire thickness) $L_{\text{end}}$, one DC‐block capacitor $C_{b}$ and 2 RF‐chokes is presented at Figure 2C. Using two series‐connected PIN diodes allows the protection of the switching unit from switching by the RF pulse itself. The switchable array was tested in transceiver mode using a home built TxRx interface consisting of eight LNAs (WantComm, USA) and eight high‐power TR‐switches [26]. A detailed electric circuit of a single switchable antenna is presented in Figure 2D. Switching time (∼20 μs) is defined by the PIN diode carrier lifetime.

### 2.5 MRI Measurements and Pulse Design

Experiments were performed on a Magnetom 9.4 T Plus whole‐body MRI scanner (Siemens Healthineers, Germany). All experiments were performed with the approval of the local ethics committee and after obtaining written informed consent. The MR sequences were written in a modified version of Pulseq [27, 28], which allows pTx RF‐shimming for each RF pulse. Single‐channel $B_{1}^{+}$ and $B_{0}$ maps were acquired using a 3D presaturated turbo‐flash sequence with weighted hybrid mapping [29]. $B_{1}^{+}$ and $B_{0}$ mapping was performed in a cylindrical phantom and in a healthy male subject. The $B_{1}^{+}$ maps were acquired for both the “up” and the “down” configuration of the switchable array. The $B_{1}^{+}$ maps of the “up” and “down” configuration and their difference were compared to the equivalent maps from the simulation.

A homogeneous flip angle distribution of 10° throughout the brain was chosen as the optimization target. The nRMSE of the obtained flip angle distribution was optimized with 20 iterations of a Tikhonov‐regularized minimization. Optimization was performed for the entire volume in the phantom and for the brain only in‐vivo, based on $B_{1}^{+}$ maps measured during the same scan session. Brain masks were generated from the proton‐density weighted reference image of the presaturated turbo‐flash sequence using SynthSeg [30]. For excitation, 2‐kT points pulses were designed. Fast switching during the pulse was implemented by changing the coil profiles from up to down configuration after half the excitation time. In this case, magnitudes and phases were optimized for each subpulse.

Similar to the simulation experiments, the k‐space location of the first kT point was chosen by randomly generating 25 000 k‐space positions in the range $-28\;m^{-1}\leq k_{x,y,z}\leq 28\;m^{-1}$ and selecting the one that gave the best performance for the given scenario. The second kT point was again positioned in the center of the k‐space.

## 3 Results

Numerically simulated $B_{1}^{+}$ maps in the central transversal plane of a homogeneous cylindrical phantom are presented in Figure 1C for the “down” and “up” cases. The ratio between these two $B_{1}^{+}$ profiles is presented at Figure 1D, clearly illustrating the effect of skewed current densities on the resulting transmit sensitivity patterns.

As expected, the $B_{1}^{+}$ field distribution varied between the “up” and “down” states of the Tx elements also when integrated into an entire coil array. When switching from the “down” to the “up” configuration, the simulated $B_{1}^{+}$ field in the inferior regions got reduced by approximately 30%, while the field in superior regions of the brain increased by a similar magnitude. In our experimental validation, this effect was also visible, but significantly lower than in the simulations. Here, the field in the inferior regions only reduced by ca. 10%. Supporting Information Figure S1 displays the simulated and measured $B_{1}^{+}$ distribution of one element in both configurations, as well as the difference between the two configurations.

**FIGURE 3:**
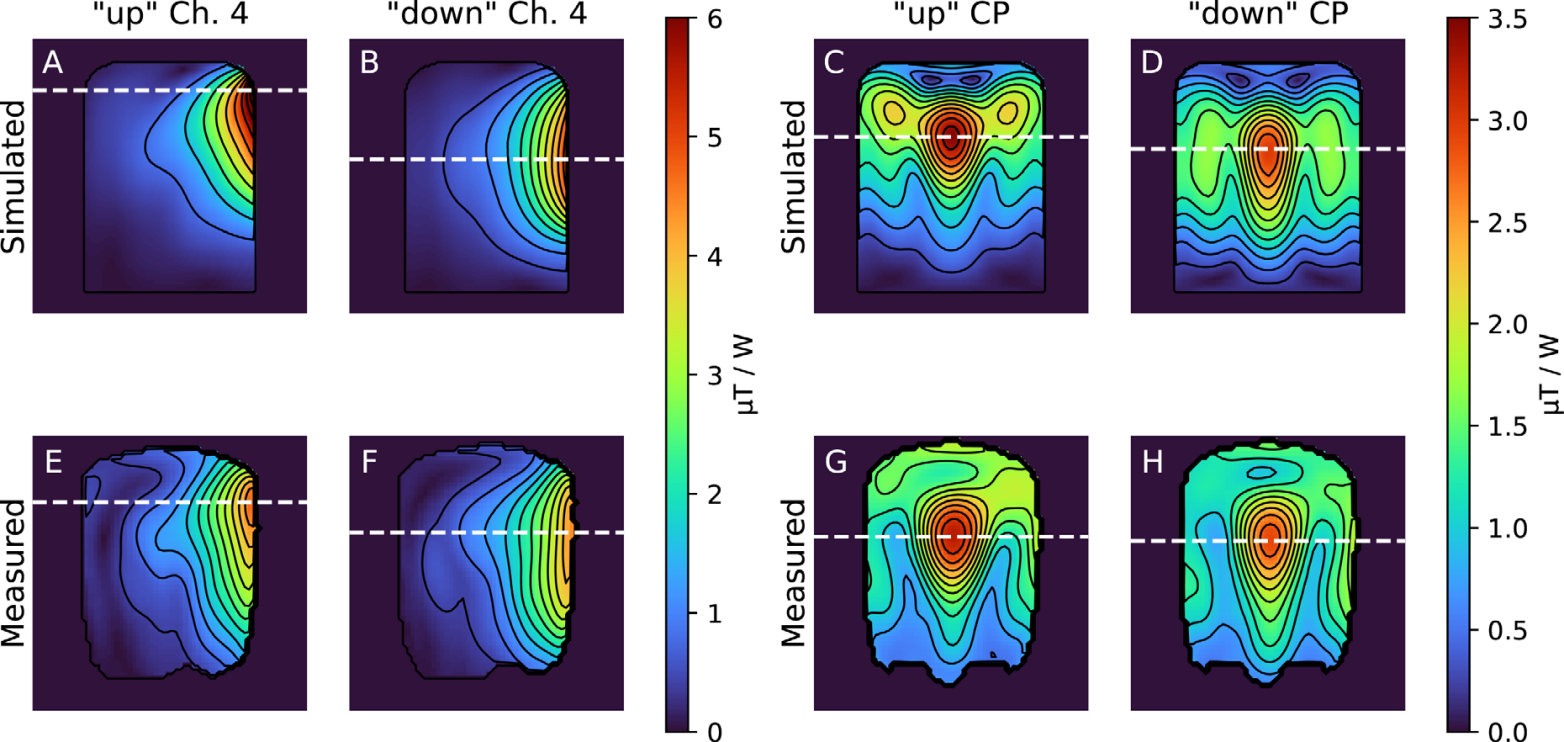
$B_{1}^{+}$ field distribution in a cylindrical phantom, from simulations (panels A–D) and in measurements (panels E–H). The dashed white line indicates the location of the peak $B_{1}^{+}$ value for each panel. In the simulated case, a single channel exhibits a strong switching behavior (Panels A and B). In the experiment (panels E and F) this effect is reduced. In CP mode simulations (panels C and D), there is still a clear shift of the $B_{1}^{+}$ field distribution when switching. In the experiment (panels G and H) this effect is barely visible.

In the cylindrical phantom, the behavior was similar (Figure 3). In the simulations, the intensity profile in CP mode is clearly shifted toward the lower end, including the location of the peak $B_{1}^{+}$ value. In the measurements, the profile shows changes from switching too, but the $B_{1}^{+}$ peak appears to barely change position.

The S‐matrix did not change significantly when changing from the “up” to the “down” configuration, suggesting that neither reflected power nor coupling between the elements increased notably when changing configuration. The exact S‐matrices can be seen in Supporting Information: Figure S2.

### 3.1 Optimized RF Pulses Based on Simulated Coil

**FIGURE 4:**
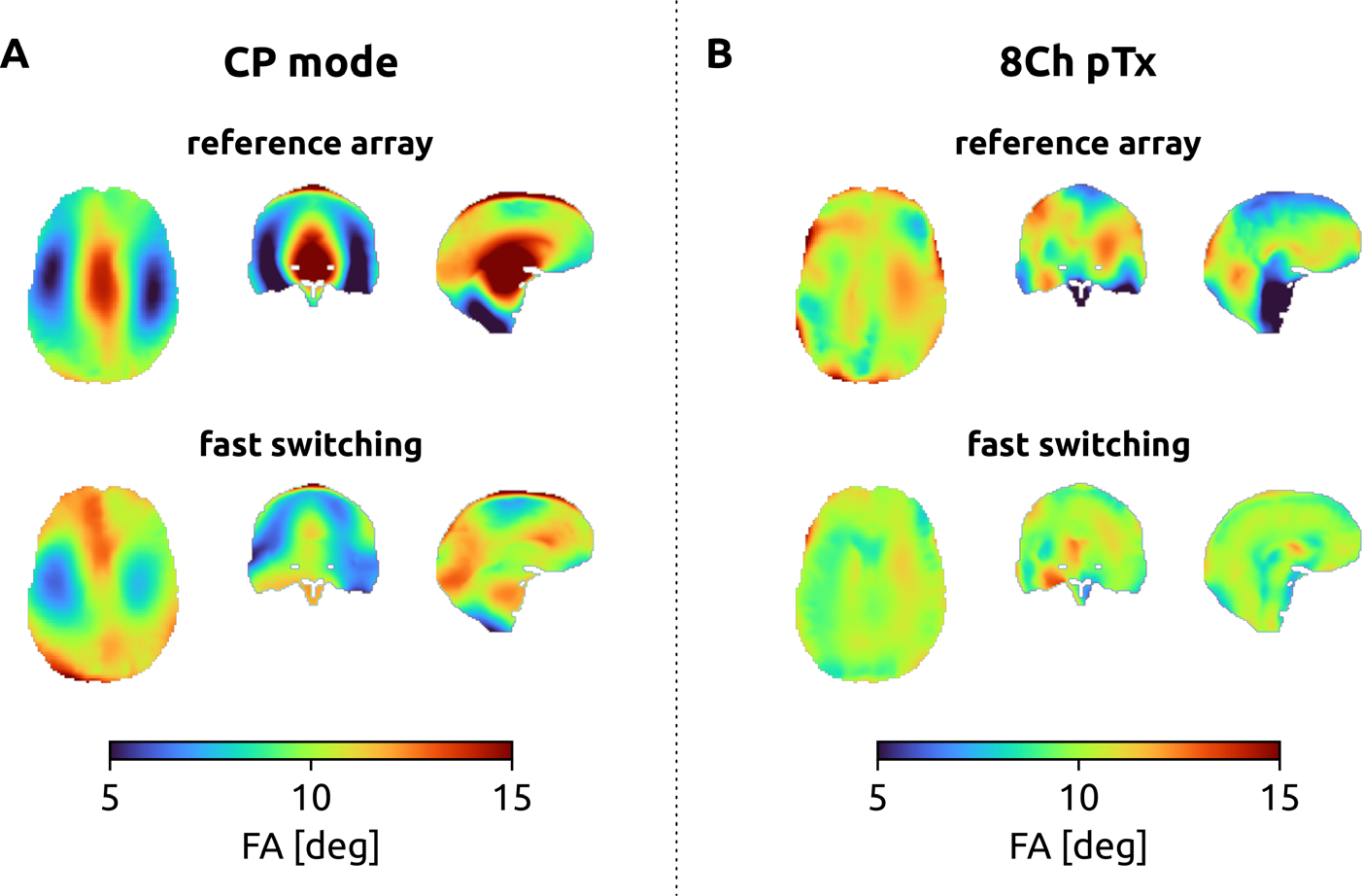
Resulting flip angle distributions from simulations for 2 kT points pulses optimized on “Duke” in **(A)** CP mode operation, and **(B)** 8 channel pTx operation.

When operating the coil in CP‐mode, switching between the two coil sensitivities after half of a single excitation pulse improved the nRMSE from 32.47% to 30.08%. When using two kT points in CP mode, switching after half of every subpulse improved the nRMSE from 28.15% to 16.75% (Figure 4A). When operating the coil in pTx mode, a single excitation pulse also benefited from fast sensitivity switching, as the nRMSE decreased from 29.47% to 19.08%. When designing a two kT pTx pulse, a nRMSE of 15.80% (reference) and 7.27% (switched) were achieved (Figure 4B).

### 3.2 Optimized RF Pulses Based on Constructed Prototype Coil

**FIGURE 5:**
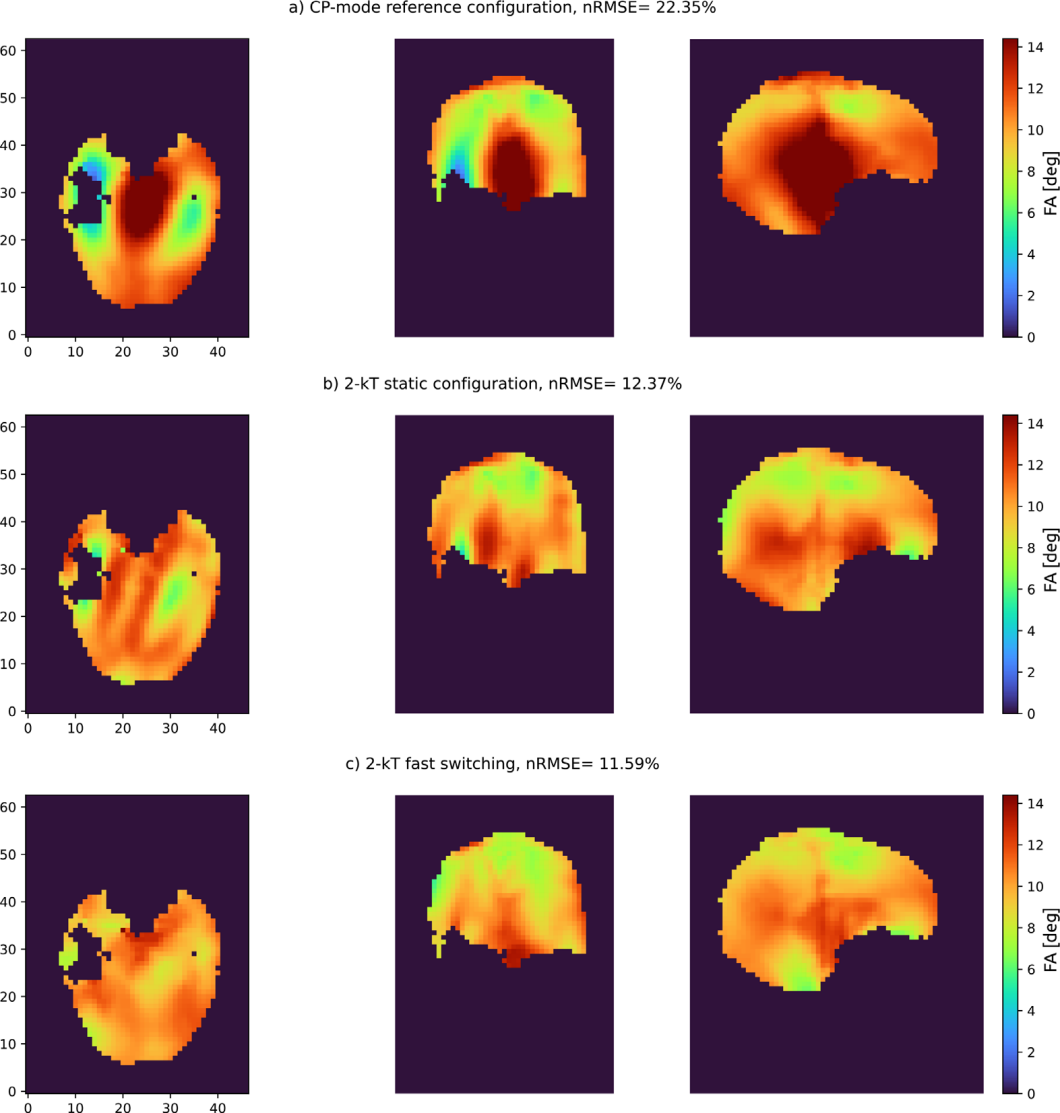
Resulting flip angle distributions from pulse optimization achieved on a subject. **(A)** Single‐pulse CP mode configuration of the reference coil. **(B)** 2 kT points (pTx) pulse for the reference coil. **(C)** 2 kT points (pTx) with fast switching of transmit sensitivities.

Based on the measured $B_{1}^{+}$ maps from the healthy volunteer, an nRMSE of 22.35% was achieved for CP mode in the reference array (Figure 5A). The 2 kT points pTx pulse for the reference array achieved an nRMSE of 12.37% (Figure 5B). Introducing sensitivity switching reduced the nRMSE to 11.59% (Figure 5C).

## 4 Discussion and Conclusion

In simulations, introducing transmit elements with rapidly switchable $B_{1}^{+}$ sensitivities allows for a significant reduction in flip angle inhomogeneity both in CP and pTx mode operation (Figure 4). However, compared to the simulation results, the constructed array prototype exhibits only a weak difference in the sensitivities of the “up” and “down” configurations, indicating that the effect of fast switching is well below expectations. In contrast, previous work on similarly designed switchable Rx coaxial dipoles demonstrated a reasonable diversity between “up” and “down” configurations for both simulations and experimental results [11].

A potential explanation for this disagreement is a suboptimal numerical model of the switchable unit. The entire switchable unit was simulated as an ideal single capacitive or inductive element. For the prototype, however, it was realized as a physical combination of different lumped elements soldered onto a PCB including relatively large PIN diodes required to switch high RF power. This addition of bulky PCB can lead to lower diversity between the “up” and “down” profiles. First, the Tx dipole length was increased (from 13 cm to 17 cm) in comparison to Rx dipoles resulted in the smaller $L_{\text{end}}$ value. Including $L_{\text{end}}$ into a relatively large PCB required a further decrease of the inductance value making it comparable with the parasitic inductance of the entire PCB unit. In turn, in Rx dipoles, which didn't require high RF power elements, the entire switchable circuit was substantially smaller. However, it is difficult to include the entire PCB in the numerical model, as this would lead to a very large simulation time. Next steps for improving upon that might be using different types of switchable elements (MEMS, MOSFETS), as well as other designs of switchable units that could reduce the size of the switchable unit itself. Alternatively, the size of the dipoles can be reduced by combining two rows of sixteen shorter dipoles [19]. This will also allow using smaller PIN diodes and increasing the $L_{\text{end}}$ value.

Still, in the course of the present work, we were able to demonstrate that the concept works not only in simulations, but in principle, it can also be realized experimentally for switching high RF power generated within the resonating Tx dipoles. Similar to the observations made with reconfigurable Rx elements [9, 10, 11], switching between different Tx sensitivity patterns of a single element can be seen as a way to effectively emulate a larger number of independent virtual Tx elements. Seeing the considerable cost and technical complexity of independent Tx channels, it becomes clear that the use of rapidly switchable $B_{1}^{+}$ sensitivities in transmit elements holds the promise to improve the degrees of freedom available for pulse design without costly modifications to the Tx chain. If the switching effect could be increased to that seen in the simulations, a large number of eight channel pTx MRI systems could potentially be upgraded with a comparatively cheap hardware addition, which could drastically improve performance in areas that now prove difficult, such as combined whole‐brain and C‐spine imaging [31].

## Funding

This work was supported by the European Research Council (Grant No. 834940) and the Deutsche Forschungsgemeinschaft (Grant No. 530130666).

## Supporting information




**Figure S1.** Simulated and measured Tx sensitivities for both the “up” and “down” case. While the switching has an effect, it is less pronounced than in the simulations.
**Figure S2.** Scattering matrix (S‐matrix) for the switchable array with a headand‐shoulder shaped phantom inside. A and B: the simulated array was tuned and matched in the “up”‐configuration. Switching to the “down”‐configuration only caused minor changes in coupling and reflection. C and D: a similar behavior was observed in a benchtop measurement.

## Data Availability

The data that support the findings of this study are available from the corresponding author upon reasonable request.
