# Supplementary material for: Coaxial Dipole Array With Switching Transmit Sensitivities for Ultrahigh Field MRI
Source: Magn Reson Med. 2026 Feb 9;95(6):3608–15. doi: 10.1002/mrm.70243 (PMC13049251; doi:10.1002/mrm.70243)
Supplement: Supplementary file 1 — Figure S1. Simulated and measured Tx sensitivities for both the “up” and “down” case. While the switching has an effect, it is less pronounced than in the simulations. Figure S2. Scattering matrix (S‐matrix) for the switchable array with a headand‐shoulder shaped phantom inside. A and B: the simulated array was tuned and matched in the “up”‐configuration. Switching to the “down”‐configuration only caused minor changes in coupling and reflection. C and D: a similar behavior was observed in a benchtop measurement. [file MRM-95-3608-s001.pdf]

Coaxial Dipole Array with Switching Transmit Sensitivities for ultrahigh field MRI

Supporting Information

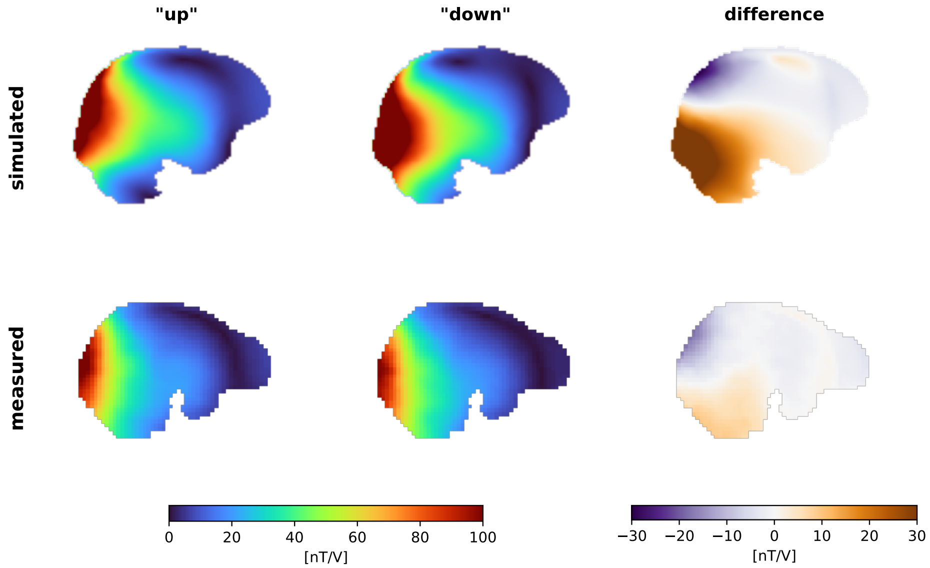

Figure S1: Simulated and measured Tx sensitivities for both the "up" and "down" case. While the switching has an effect, it is less pronounced than in the simulations.

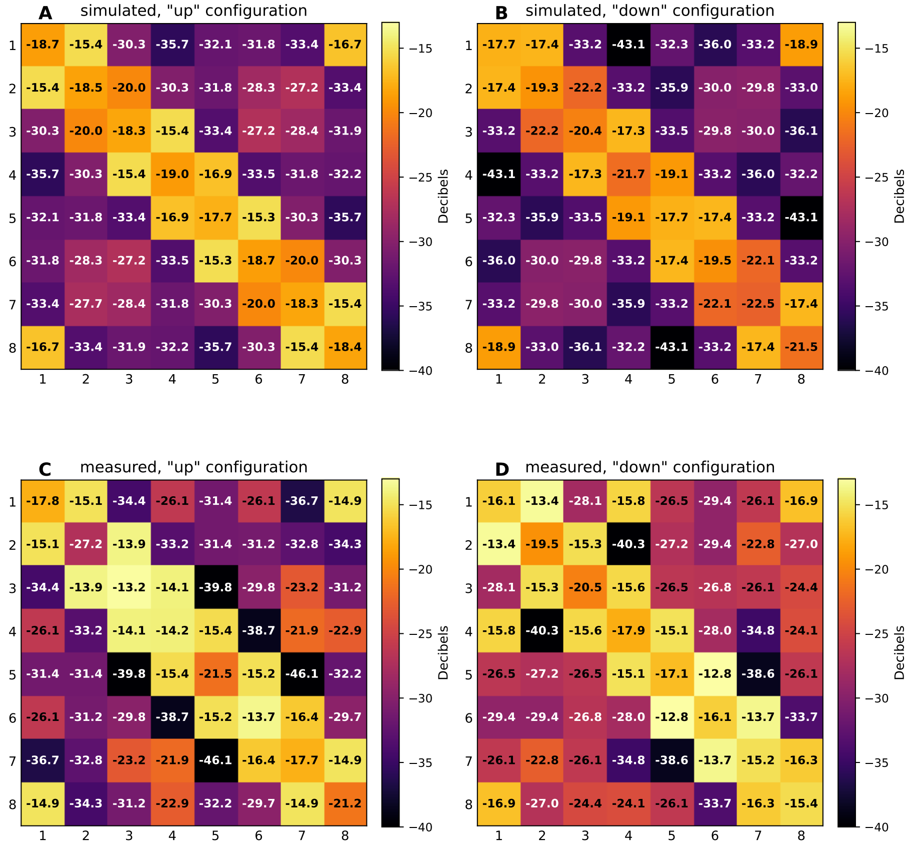

Figure S2: Scattering matrix (S-matrix) for the switchable array with a head-and-shoulder shaped phantom inside. A and B: the simulated array was tuned and matched in the "up"-configuration. Switching to the "down"-configuration only caused minor changes in coupling and reflection. C and D: a similar behavior was observed in a benchtop measurement.
